# Impact of Telemedicine Use by Oncology Physicians on the Patient and Informal Caregiver Experience of Receiving Care: Protocol for a Scoping Review in the Context of COVID-19

**DOI:** 10.2196/25501

**Published:** 2020-12-15

**Authors:** Maclean Thiessen, Andrea Michelle Soriano, Hal John Loewen, Kathleen Margaret Decker

**Affiliations:** 1 Research Institute of Oncology and Hematology CancerCare Manitoba Winnipeg, MB Canada; 2 Department of Internal Medicine Rady Faculty of Health Sciences University of Manitoba Winnipeg, MB Canada; 3 Faculty of Nursing University of Calgary Calgary, AB Canada; 4 Max Rady College of Medicine Rady Faculty of Health Sciences University of Manitoba Winnipeg, MB Canada; 5 Neil John Maclean Health Science Library University of Manitoba Winnipeg, MB Canada; 6 Department of Community Health Sciences Rady Faculty of Health Sciences University of Manitoba Winnipeg, MB Canada

**Keywords:** cancer, experience, information needs, telemedicine, telehealth, COVID-19, patient satisfaction

## Abstract

**Background:**

During the COVID-19 pandemic, the use of telemedicine by oncology physicians in Manitoba, Canada, has increased to limit the risk of exposure to the virus for both patients and health care providers. It is not clear how telemedicine impacts the information needs of patients or the experience of receiving cancer care.

**Objective:**

The objective of this study is to describe how the use of telemedicine impacts the information needs and experience of patients with cancer and their informal caregivers (ie, family and friends) and identify directions for future research.

**Methods:**

This review will include all studies addressing telemedicine in the cancer context including those using quantitative, qualitative, and mixed methods approaches. This scoping review will be conducted using the methodology described by the Joanna Briggs Institute. In collaboration with a librarian scientist specializing in health sciences, a comprehensive search will be undertaken to identify and retrieve relevant reports published in English from 1990 to the present. Databases searched will include MEDLINE, CINAHL, EMBASE, Scopus, Cochrane Library, and PsycINFO. Data will be extracted by two independent reviewers, synthesized, and reported in a summary table and in a narrative format describing what has been reported regarding the impact of telemedicine by physicians in oncology on the experience of patients and their informal caregivers and their receipt of information.

**Results:**

The results from this scoping review are expected to be available by late spring 2021.

**Conclusions:**

The results from this scoping review will be useful for informing practice as well as directing future research, both in the context of COVID-19 and beyond.

**International Registered Report Identifier (IRRID):**

PRR1-10.2196/25501

## Introduction

The COVID-19 pandemic has transformed how cancer care is delivered. In the Canadian province of Manitoba, most cancer care is delivered by a provincial, centralized agency, CancerCare Manitoba (CCMB). During the COVID-19 pandemic, CCMB implemented changes to mitigate the risk of contracting the virus for patients with cancer as well as health care providers. These changes have included routine screening for signs of infection prior to staff, patients, and informal caregivers entering facilities, limiting the number of informal caregivers that can accompany patients to appointments and procedures, and the routine use of personal protective equipment (PPE) by health care staff who provide in-person care to patients [1]. Perhaps the most remarkable change in clinical practice has been the dramatic increase in the use of telemedicine by physicians.

Telemedicine and telehealth are closely related but distinct concepts [2,3]. The American Academy of Family Practitioners (AAFP) provides clear definitions of these terms [3]. The AAFP defines telehealth as the use of telecommunication strategies to deliver health care and health care–related services such as the education of health care professionals. Telemedicine is more narrowly defined and refers specifically to the delivery of medical care using telecommunications, suggesting that it is a distinct entity within the umbrella of telehealth. It should be noted that the distinction between the two terms is not recognized by all, as the terms telemedicine and telehealth have been used interchangeably by some authors [4].

In Manitoba, a provincial telehealth service has been long-standing, which facilitates telemedicine services through physician-to-patient videoconferencing services. In the cancer setting, these services link specialists working in the urban cancer centers (ie, in the cities of Winnipeg and Brandon, Manitoba) with patients attending appointments in rural and remote centers throughout the province. In response to the COVID-19 pandemic, CCMB physicians rapidly adopted telemedicine solutions outside of the formal Telehealth service. Microsoft Teams, Zoom, and other consumer-level videoconferencing services, in addition to the increased use of telephone calls, have been used in lieu of clinic visits to keep patients, informal caregivers, and health care providers safe by limiting traffic in health centers and reducing the need for in-person clinic visits.

In cancer care, physicians are often required to share sensitive and complex information with patients and those supporting them. Information needs are among the most commonly cited unmet supportive care needs [5,6], and it is not clear how receiving information, including that which is considered to be “bad news,” through telemedicine impacts the patient experience. With the rapid increase of telemedicine services during the COVID-19 pandemic, including the use of informal telemedicine services in Manitoba, it is necessary to better understand how telemedicine affects the experience of receiving information from physicians.

Telemedicine and telehealth have been explored outside of the cancer setting, with numerous intervention trials and systematic reviews both completed and underway [7]. Additionally, guidelines exist to help guide best practice for the use of telemedicine [7], such as those developed by the American Association of Telemedicine [8]. However, in the unique setting of oncology, there appears to be a lack of empirical evidence to guide best practices. Prior to undertaking this review, the literature was searched to identify literature that would be helpful for guiding best practices. The search included several databases (JBI Evidence, Cochrane Systemic Reviews, and MEDLINE) and did identify numerous studies that have explored the experience of receiving cancer care through telemedicine, with two reviews identified that summarized the qualitative evidence regarding the experience of patients with cancer and informal caregivers accessing telemedicine [9,10]. However, no review could be identified that addressed specific activities or practices oncology physicians could employ to improve the experience of receiving care through telemedicine. Of note, a recent publication on delivering bad news using telemedicine in the oncology setting based on expert opinion guided by the SPIKES framework [11] was identified, but it is noted that this framework was not specifically designed in the context of telemedicine.

Systematic, scoping, and mapping reviews are 3 different types of systematic literature review methods, each with distinct procedures and outputs. Systematic reviews are useful when a decision regarding changes to current practice is required [12]. Scoping reviews, which may be a precursor to systematic reviews, help to identify the types of evidence available on a given topic, clarify concepts, examine how research has been conducted, and identify knowledge gaps [12,13]. Mapping reviews, on the other hand, do not answer specific questions about a topic, but identify what evidence exists regarding a certain topic [14,15]. As the literature relevant to the required objectives does not appear to have been previously summarized, a scoping review was selected as the best review strategy.

Based on how telemedicine has been adopted at CCMB during the COVID-19 pandemic, the relevant objectives for this review are the following: (1) to identify how telemedicine in the oncology setting has been studied in the literature, (2) to identify what specific clinician practices have either been demonstrated or suggested to be helpful in terms of improving the patient/friend/family experience, and (3) to identify future research directions to improve the integration of telemedicine with clinical oncology care.

## Methods

This scoping review protocol is based on the methodology developed by the Joanna Briggs Institute (JBI) [12,16]. In keeping with their procedures, the following section will outline the specific review questions, study inclusion criteria, search strategy, and procedures for study selection, data extraction, and data presentation.

### Review Questions

The primary question this review will seek to answer is the following:

Question 1.0: What studies have been conducted examining the relationship between clinicians’ use of telemedicine and the experience of patients with cancer and their informal caregivers?

The subquestions this review will seek to answer are the following:

Question 1.1: What clinician factors have been identified as impacting the experience of receiving care via telemedicine in the cancer context?

Question 1.2: What patient/informal caregiver factors have been identified as affecting the experience of receiving care via telemedicine in the cancer context?

Question 1.3: What types of communication strategies have been studied for delivering telemedicine in the cancer context?

Question 1.4: What technological factors (eg, apps, remote monitoring, asynchronous versus synchronous communication) have been demonstrated to impact the experience of receiving care via telemedicine for patients and their informal caregivers?

Question 1.5: What factors related to the type of information being shared have been demonstrated to impact the cancer experience of patients and their informal caregivers?

### Inclusion Criteria

#### Participants

This review will include studies involving patients with cancer aged >18 years and their friends, family, and informal caregivers.

#### Concept

Any telemedicine use in the oncology context.

#### Context

The studies included in this review will focus on the receipt of oncology physician care through telemedicine. This review will not focus on specific cultural, racial, or geographic characteristics.

#### Types of Sources

This scoping review will consider quantitative, qualitative, and mixed methods study designs for inclusion. Additionally, systematic reviews and editorials will be considered for inclusion. Articles published in English will be included. Articles published from 1990 to the present will be included to reflect research conducted at the beginning of the contemporary era of telemedicine [17].

### Exclusion Criteria

Studies focused primarily on the pediatric oncology context and non–peer reviewed publications will be excluded.

### Search Strategy

The search strategy will aim to locate peer-reviewed published primary studies, reviews, and editorials. An initial limited search of MEDLINE and CINAHL was undertaken to identify articles on the topic. The text words contained in the titles and abstracts of relevant articles, and the index terms used to describe the articles, were used to develop a full search strategy for MEDLINE, and translated for CINAHL (see Multimedia Appendix 1 for the MEDLINE search). The search strategy, including all identified keywords and index terms, will be adapted for each included information source. The reference lists of articles selected for full-text review will be screened for additional papers.

### Information Sources

Information sources will include electronic databases and study authors. Databases to be searched will include MEDLINE, CINAHL, EMBASE, Cochrane Library, Scopus, and PsycINFO.

### Study Selection

Following the search, all identified records will be collated and uploaded into EndNote X9 (Clarivate Analytics) and duplicates will be removed. Titles and abstracts will then be screened by two independent reviewers for assessment against the inclusion criteria for the review. Potentially relevant papers will be retrieved in full and their citation details will be imported into an Excel (Microsoft Corp) spreadsheet. The full text of selected citations will be assessed in detail against the inclusion criteria by two independent reviewers. Reasons for exclusion of full-text papers that do not meet the inclusion criteria will be recorded and reported in the scoping review. Any disagreements that arise between the reviewers at each stage of the selection process will be resolved through discussion or with a third reviewer. The results of the search will be reported in full in the final scoping review and presented in a PRISMA-ScR (Preferred Reporting Items for Systematic Reviews and Meta-analyses Extension for Scoping Reviews) flow diagram [18].

### Data Extraction

Data will be extracted from papers included in the scoping review by two independent reviewers using a data extraction tool developed by the reviewers. The data extracted will include specific details about studies exploring the experience of adult patients with cancer and their informal caregivers receiving telemedicine relevant to the main review question as well as the scoping review subquestions. The extraction tool has been developed based on recommendations and the example provided by JBI [19]. A draft extraction tool is provided (Multimedia Appendix 2). The draft data extraction tool will be modified and revised as necessary during the process of extracting data from each included paper. Modifications will be detailed in the full scoping review. Any disagreements that arise between the reviewers will be resolved through discussion or with a third reviewer. Authors of papers will be contacted to request missing or additional data, where required.

### Data Presentation

The extracted data will be presented in diagrammatic or tabular form in a manner that aligns with the objective of this scoping review. A narrative summary will accompany the tabulated and/or charted results and will describe how the results relate to the review’s objectives and questions.

## Results

Study activities related to the scoping review will begin in December 2020. Results will be available by late spring 2021.

## Discussion

The protocol for this scoping review has been developed as a pragmatic response to the challenges faced by patients, their informal caregivers, and clinicians in accessing/providing clinical care during the global COVID-19 pandemic. The need to socially distance and take additional precautions to prevent transmission of the COVID-19 virus has likely had significant effects on the experience of receiving and delivering [20] cancer care. How these changes have impacted the delivery and receipt of information provided by clinicians to patients and their informal caregivers is not yet fully understood.

A recent report by the lead author of this protocol (MT) identified key characteristics of high-quality information. These emerged from semistructured interviews with 60 patients with cancer and their friends and family [21]. The interview data were analyzed using Classical Grounded Theory [22-24]. The key characteristics of high-quality information included accessibility, credibility, applicability, and framing. Accessibility was defined as the relative convenience and effort associated with receiving information about cancer. Credibility reflected how trustworthy/reliable the information was considered. Applicability referred to the degree with which the information received applied to the individual who received it. Framing was defined as how the information was presented, with particular focus on whether the information identified ways that the cancer situation could be optimized, either by clinicians or by the individuals receiving the information. Using the framework provided by this previous study [21], both from a patient and informal caregiver perspective, receiving care through telemedicine may be more accessible than through traditional in-person encounters. However, some important information conveyed through in-person interactions maybe not be transmitted. For instance, nonverbal information may not be delivered or received, making it challenging to both provide and receive care in sensitive contexts. This scoping review will be useful in identifying the different factors that have been found to affect the delivery of information using telemedicine in the cancer context as well as what areas have yet to be explored. The results will be useful to both clinicians and researchers in the context of the COVID-19 pandemic and beyond.

## Appendix

Multimedia Appendix 1 Search strategy.

Multimedia Appendix 2 Data extraction instrument.

## Abbreviations

AAFP: American Academy of Family Practitioners
CCMB: CancerCare Manitoba
JBI: Joanna Briggs Institute
PPE: personal protective equipment
PRISMA-ScR: Preferred Reporting Items for Systematic Reviews and Meta-analyses Extension for Scoping Reviews
