# Supplementary material for: Impact of Telemedicine Use by Oncology Physicians on the Patient and Informal Caregiver Experience of Receiving Care: Protocol for a Scoping Review in the Context of COVID-19
Source: JMIR Res Protoc. 2020 Dec 15;9(12):e25501. doi: 10.2196/25501 (PMC7744149; doi:10.2196/25501)
Supplement: Multimedia Appendix 1 [file resprot_v9i12e25501_app1.docx]

**Appendix I: Search strategy.**

OVID MEDLINE October 30 2020

| **#** | **Searches** | **Results** |
| --- | --- | --- |
| 1 | exp Neoplasms/ or Bone Marrow Transplantation/ or exp Stem Cell Transplantation/ or exp Antineoplastic Agents/ or exp Immune Tolerance/ or Neutropenia/ or (cancer$ or neoplas$ or tumor$ or tumour$ or malignan$ or carcinoma$ or metasta$ or masses or oncolog$ or leukemi$ or leukaemi$ or lymphoma$ or myeloma$ or sarcoma$ or chemotherap$ or transplant$ or HSCT or BMT or neutropenia or immuno-compromise$ or h?ematolog$ or immunocompromise$ or radiation or "proton therap$" or ((lymphocyte$ adj5 (function$ or count$ or number$)) or ((immuno adj1 suppress$) or immunosuppress$) or ((immunity or immune) adj5 (suppress$ or impair$ or dysfunction$)))).tw,kf. | 5812202 |
| 2 | physicians/ or "Internship and Residency"/ or (physician$ or doctor$ or resident$ or allergist$ or an?esthesiologist$ or cardiologist$ or clinician? or dermatologist$ or endocrinologist$ or "foreign medical graduate$" or hepatologist or geriatrician$ or gastroenterologist$ or gerontologist$ or hospitalist or intern or intern or internship$ or nephrologist$ or neurologist$ or neurosurgeon$ or oncologist$ or ophthalmologist$ or osteopath$ or otolaryngologist$ or otologist$ or pathologist$ or pediatrician or neonatologist$ or physiatrist$ or pulmonologist$ or radiologist$ or residenc$ or rheumatologist$ or surgeon$ or urologist$ or ((family or general) adj2 practioner$)).tw,kf. | 1352334 |
| 3 | exp telemedicine/ or videoconferencing/ or telecommunications/ or telephone/ or cell phone/ or (((remote or distance or electronic or mobile or computer or screen) adj3 (consult$ or interveiw$ or monitor$ or care or follow-up)) or (mobile adj3 (health or care or healthcare or medicine or medical)) or (app? or synchronous or asynchronous or a-synchronous or telehealth or telemedicine or tele-medicine or ehealth or e-health or mhealth or m-health or telepathology or realtime or real-time or "real time" or tele-pathology or teleradiology or tele-radiology or telerehabilitation or tele-rehabilitation or videoconferenc* or telecommunication$ or teleconference$ or telephone$ or store-forward or "store forward") or ((telephone$ or phone$) adj3 (cell or cellular or mobile)) or ((online or internet) adj3 meeting$)).tw,kf. | 478238 |
| 4 | exp Patient Satisfaction/ or ((patient* or client*) adj3 (happiness or experienc* or feeling* or satisfaction or comfort* or response or disappoint* or displeasure or disappoint* or unhapp* or un-happ*)).tw,kf. | 312985 |
| 5 | 1 and 2 and 3 and 4 | 477 |
