# Supplementary material for: Impact of Telemedicine Use by Oncology Physicians on the Patient and Informal Caregiver Experience of Receiving Care: Protocol for a Scoping Review in the Context of COVID-19
Source: JMIR Res Protoc. 2020 Dec 15;9(12):e25501. doi: 10.2196/25501 (PMC7744149; doi:10.2196/25501)
Supplement: Multimedia Appendix 2 [file resprot_v9i12e25501_app2.docx]

**Appendix II: Data extraction instrument.**

| Title |  |
| --- | --- |
| Authors |  |
| Year of Publication |  |
| Country were study conducted |  |
| Aims |  |
| Study population |  |
| sample size |  |
| methods |  |
| Intervention Type |  |
| Comparator (control) |  |
| Duration of intervention |  |
| Outcomes and details of these |  |
| Findings related to Q1.0 |  |
| Findings related to Q1.1 |  |
| Findings related to Q1.2 |  |
| Findings related to Q1.3 |  |
| Findings related to Q1.4 |  |
| Findings related to Q1.5 |  |
